# Development of Precipitation-Strengthened Al_0.8_NbTiVM (M = Co, Ni) Light-Weight Refractory High-Entropy Alloys

**DOI:** 10.3390/ma14082085

**Published:** 2021-04-20

**Authors:** Kangjin Lee, Yunjong Jung, Junhee Han, Sung Hwan Hong, Ki Buem Kim, Peter K. Liaw, Chanho Lee, Gian Song

**Affiliations:** 1 Division of Advanced Materials Engineering and Institute for Rare Metals, Kongju National University, Cheonan, Chungnam 330-717, Korea; lkjlkm22@naver.com (K.L.); koer080@naver.com (Y.J.); 2 Korea Institute for Rare Metals, Korea Institute of Industrial Technology, 7-47 Songdo-dong Yeonsoo-gu, Incheon 406-840, Korea; jhan@kitech.re.kr; 3 Faculty of Nanotechnology and Advanced Materials Engineering, Sejong University, Gwangjin-gu, Seoul 143-747, Korea; shhong@sejong.ac.kr (S.H.H.); kbkim@sejong.ac.kr (K.B.K.); 4 Department of Materials Science and Engineering, The University of Tennessee, Knoxville, TN 37916, USA; pliaw@utk.edu; 5 Materials Science and Technology Division, Los Alamos National Laboratory, Los Alamos, NM 87545, USA

**Keywords:** high-entropy alloys, refractory alloys, lightweight, sigma phase, mechanical properties

## Abstract

Single-phase solid-solution refractory high-entropy alloys (RHEAs) have been receiving significant attention due to their excellent mechanical properties and phase stability at elevated temperatures. Recently, many studies have been reported regarding the precipitation-enhanced alloy design strategy to further improve the mechanical properties of RHEAs at elevated temperatures. In this study, we attempted to develop precipitation-hardened light-weight RHEAs via addition of Ni or Co into Al_0.8_NbTiV HEA. The added elements were selected due to their smaller atomic radius and larger mixing enthalpy, which is known to stimulate the formation of precipitates. The addition of the Ni or Co leads to the formation of the sigma precipitates with homogeneous distribution. The formation and homogeneous distribution of sigma particles plays a critical role in improvement of yield strength. Furthermore, the Al_0.8_NbTiVM_0.2_ (M = Co, Ni) HEAs show excellent specific yield strength compared to single-phase AlNbTiV and NbTiVZr RHEA alloys and conventional Ni-based superalloy (Inconel 718) at elevated temperatures.

## 1. Introduction

High-entropy alloys (HEAs) are defined as alloys that are composed of more than 4 elements with near equi-atomic percentage [1,2,3,4]. Due to their high mixing entropy, HEAs tend to form simple solid-solution phases, such as face-centered-cubic (FCC) [5,6], body-centered-cubic (BCC) [7,8,9], and hexagonal-close-packed (HCP) structures [10,11]. With the entropic stabilization of solid-solution phases, HEAs have attracted significant attention, because of their outstanding properties at elevated temperatures, which results from severe lattice distortion and microstructural stability at high temperatures [9,12,13].

Many refractory HEAs (RHEAs), which mainly consist of BCC-type single solid-solution phase, have been developed and investigated due to their excellent mechanical properties at elevated temperatures [14]. The excellent mechanical properties at high temperatures in RHEAs are significantly associated with their unique atomic structures (distorted lattices) and high melting temperatures of the constitutive refractory elements (Ti, Nb, V, Mo, Ta, W, and Cr) [7,8,9,14]. However, mechanical properties of the single solid-solution RHEAs at elevated temperature need to be further improved by a new alloy design approach for the high-temperature applications in aerospace and aviation industries, improving the strength, thermal stabilities, and density.

To meet the increasing demand of high-temperature alloys, there have been many efforts to design the RHEAs through the new alloy design approaches [15,16,17,18,19]. For example, RHEAs reinforced by formation of secondary phases, such as precipitates, which results in considerable improvement of the yield strength at high temperatures [17,20]. The formation of second phases in the RHEAs can be anticipated by using mixing enthalpy difference between the alloying elements, i.e., the large negative mixing enthalpy favors the formation of intermetallic phase [21]. In addition, it has been reported that the large atomic size difference between the composed elements can lead to formation of intermetallic phases [22]. For instance, the Cr induces the formation of laves phase due to its large difference of atomic radius among the composed elements in the Al-Cr-Nb-Ti-V and Cr-Nb-Ti-V-Zr systems [17,23]. The laves phases in the Al-Cr-Nb-Ti-V and Cr-Nb-Ti-V-Zr RHEAs lead to improvement of yield strength at 800 °C compared to the single solid-solution RHEAs [17,23].

In our present study, we attempted to design the new light-weight RHEAs adjusting the amount of Al content in AlxNbTiV alloys (X = 0, 0.4, 0.8, and 1). It is found that present RHEAs exhibit maintenance of single BCC phase formation with different Al contents and the Al_0.8_NbTiV shows the combination of high yield strength and good plasticity. Further addition of Ni or Co was made (which has larger mixing enthalpy than other constitutive alloying elements) to stimulate the formation of precipitations. It was found that the addition of Ni or Co successfully induced the formation of sigma precipitates in the BCC matrix, and it results in the enhancement of the mechanical properties at high temperatures up to 700 °C.

## 2. Experimental Methods

The alloy ingots were fabricated by using vacuum-arc-melting with Ti, Nb, V, Al, Co, and Ni elements of 99.95 wt.% elemental purity under a Ti-gettered high-purity argon atmosphere (99.99%). The ingots were solidified on a water-cooled copper hearth, and the ingots were re-melted more than 8 times by flipping them over to achieve homogenous distribution of elements. The 8 times remelting process has been conducted in an arc-melting chamber after initial melting of elements. The as-cast rod samples were produced by suction casting into a cylindrical Cu mold with 3 mm in diameter and 50 mm in length. The nominal compositions of the alloys are listed in Table 1.

After casting, the fabricated Al-Nb-Ti-V-M (M = Co, Ni) alloys were heat-treated at 1100 °C for 1 h, using a Protherm PLF 140 box furnace (Ankara, Turkey). The samples were sealed by vacuumed (10^−2^ Torr) quartz tubes filled with titanium chips to prevent oxidation, followed by water quenching. The heat-treatment condition was carefully adjusted by trial-and-error processes. It was found that the optimized heat-treatment condition for present HEAs to achieve the excellent mechanical properties as well as homogenous distribution of precipitates is 1100 ℃ for 1 h.

Density of the heat-treated alloys was measured via hydrostatic weighting, and Table 2 summarizes the measured densities. The microstructure was analyzed using scanning electron microscopy (SEM), a TESCAN Mira LMH (TESCAN, Brno, Czech Republic) equipped with a ZEISS YAG backscattered electron detector (BSED) (Carl Zeiss, Jena, Germany), and a BRUKER X-Flash4010 energy-dispersive X-ray spectroscopy (EDS) detector (Bruker, Billerica, MA, USA). The crystal structures of the as-cast and heat-treated alloys were characterized by X-ray diffraction (XRD) using a RIGAKU diffractometer (Tokyo, Japan) and Cu-Kα radiation (λ = 1.54187 Å). Compressive mechanical tests at temperatures of 22, 700, and 800 °C with a strain rate of 1 × 10^−3^ s^−1^ were performed by Universal Test Machine (UTM), MTDI MINOS-F (MTDI, Daejeon, Korea). The size of mechanical test samples is cylindrical specimen with an aspect ratio of 2:1 (3 mm diameter and 6 mm length).

## 3. Results and Discussion

### 3.1. Microstructure and Mechanical Properties of the As-Cast AlxNbTiV (X = 0, 0.4, 0.8, 1) Alloys

**Figure 1:**
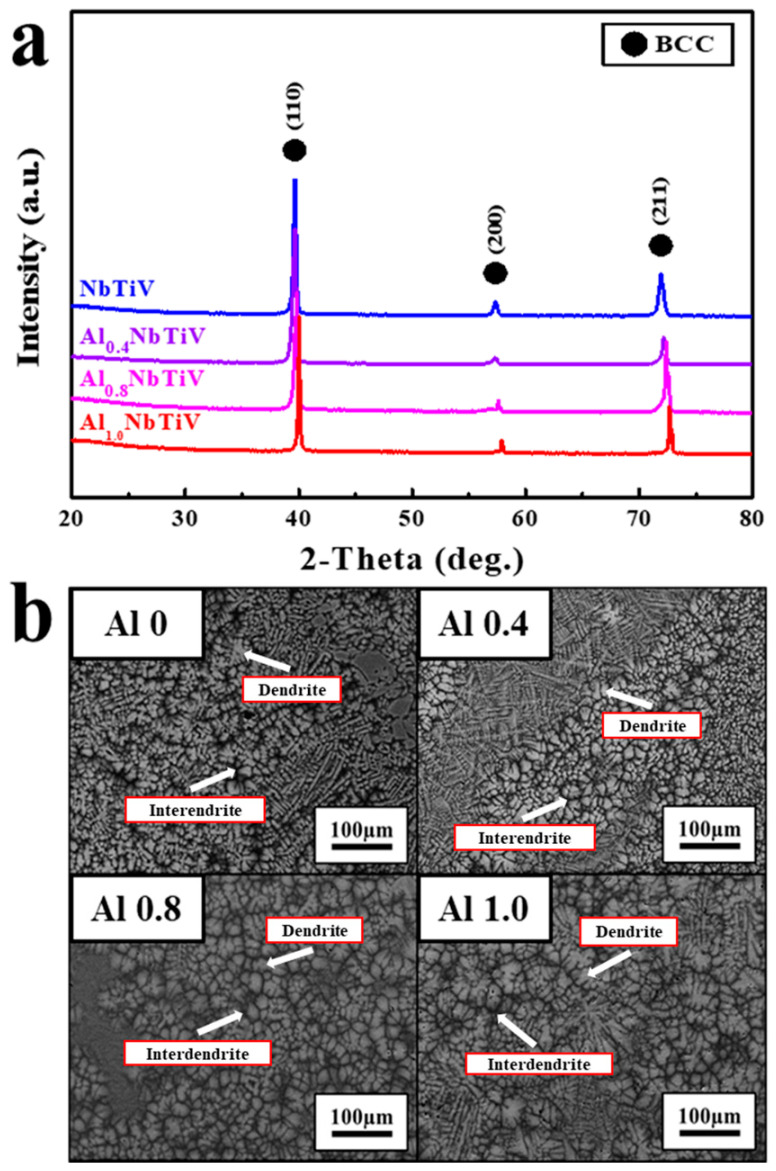
Microstructure of the as-cast Al_X_NbTiV alloys: (**a**) XRD patterns, (**b**) SEM-BSE images.

Figure 1a shows the X-ray diffraction (XRD) patterns of the as-cast AlxNbTiV (X = 0, 0.4, 0.8, and 1) alloys.

The main diffraction peaks in XRD patterns of the all-present alloys are identified as a single body-centered-cubic (BCC) structure. The lattice constants of four alloys are listed in Table 2. Note that the lattice constants of present HEAs were obtained from XRD patterns with fitting analyses.

The single BCC phase remains, and the positions of diffraction peaks are gradually shifted to the higher degree when the addition of Al is increased to 25 at.%. With the significant peak shifting, the lattice constants of BCC phase were gradually reduced from 0.3214 (NbTiV) to 0.3182 nm (AlNbTiV). The SEM-BSE images of the as-cast AlxNbTiV (X = 0, 0.4, 0.8, 1) alloys are presented in Figure 1b. All alloys indicate the typical dendritic microstructure without any noticeable changes of microstructural morphology. The chemical compositions of dendritic (bright contrast) and inter-dendritic (dark contrast) regions were examined by energy dispersive X-ray spectroscopy (EDS) line analysis, which are summarized in Table 3. The dendrites are enriched with Nb, while Al and Ti are preferentially distributed in the inter-dendrite region, indicating the chemical inhomogeneity in the as-cast condition. From the results of phase identifications and microstructures above, one can conclude that Al contents in AlxNbTiV RHEAs do not strongly influence the variation of phase formation and the features of microstructure.

**Figure 2:**
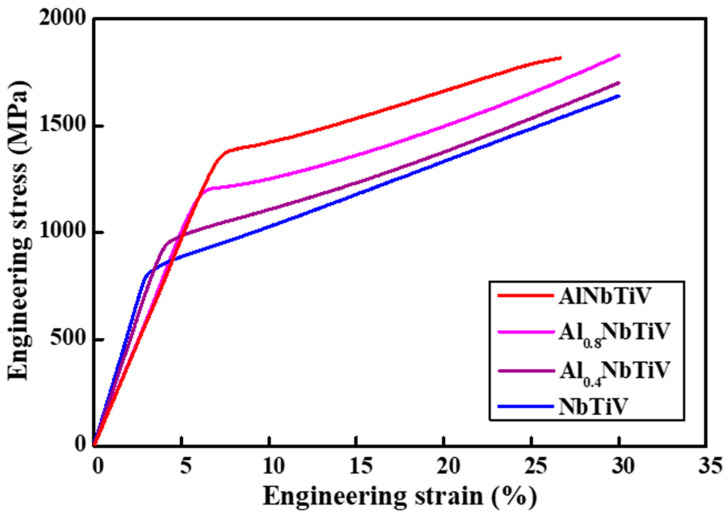
Stress–strain curves of as-cast Al_X_NbTiV (X = 0, 0.4, 0.8, 1) alloys at room temperature.

The compressive stress–strain curves of the as-cast AlxNbTiV (X = 0, 0.4, 0.8, 1) alloys at room temperature with a strain rate of 10^−3^ s^−1^ are shown in Figure 2.

The yield strength of the present alloys gradually increases as a function of Al contents, from 790 (NbTiV) to 1379 MPa (AlNbTiV). The compressive plasticity of the NbTiV, Al0.4NbTiV, and Al0.8NbTiV alloys are higher than 30% (no fracture happens until 30%, the compression tests were terminated at 30%), whereas that of the AlNbTiV alloy decreased to 20.9%. The quantitative values of yield strength (σ_y_) and compressive plastic strain (ε_p_) are listed in Table 2. The previous report on the AlNbTiV alloy subjected to homogenization heat treatment also exhibited a similar mechanical characteristic (1020 MPa of yield strength and 5% of compressive plasticity) [19]. According to the mechanical properties of present alloys, we selected Al0.8NbTiV, which shows the best combination of high yield strength, good plasticity, and low density, as a prototype alloy for further design of multi-phase RHEAs. Previously, Stepanov et al. reported the microstructural evolution and mechanical properties of multi-phase AlCrxNbTiV HEAs [17]. They found that the Cr element efficiently induces the formation of laves phases within the BCC matrix, which is probably due to the large difference of atomic radius among the composed elements [17,22]. The laves phase leads to secondary phase-strengthening and it results in improvement of yield strength at 800 °C compared to the single solid-solution RHEAs. To apply this reported alloy design approach, Co or Ni was added into the present Al0.8NbTiV alloy and the microstructure and mechanical properties were investigated, which is shown in the following section.

### 3.2. Microstructure and Mechanical Properties of Al_0.8_NbTiVM_0.2_ (M = Co, Ni) As-Cast Alloys

**Figure 3:**
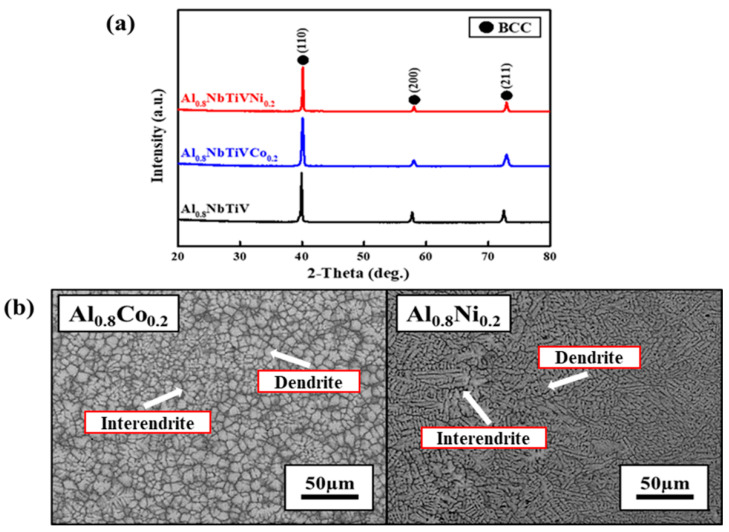
Microstructure of the as-cast Al_0.8_NbTiVM_0.2_ (M = Co, Ni) alloys: (**a**) XRD patterns, (**b**) SEM-BSE images.

Figure 3a shows the XRD patterns of the as-cast Al_0.8_NbTiVM_0.2_ (M = Co, Ni) alloys.

The XRD patterns for all studied alloys reveal that addition of miner Co and Ni elements does not play a role in secondary phase formation in Al_0.8_NbTiV alloy, exhibiting the maintenance of single BCC phase formation. The lattice constants of the BCC phase in the Al_0.8_NbTiVCo_0.2_ and Al_0.8_NbTiVNi_0.2_ as-cast alloys are calculated to be 0.31742 and 0.31738 nm respectively, which is slightly smaller than that of the Al_0.8_NbTiV alloy. Figure 3b shows the SEM image of the as-cast Al_0.8_NbTiVCo_0.2_ and Al_0.8_NbTiVNi_0.2_ alloys. Similar to the trend of phase formation, there is no noticeable change of microstructure compared to the Al_0.8_NbTiV alloy (Figure 1b). The results of EDS chemical analysis indicate that the dendrite areas are enriched with Nb, and the inter-dendrite areas are enriched with M (Co, Ni) and Ti.

**Figure 4:**
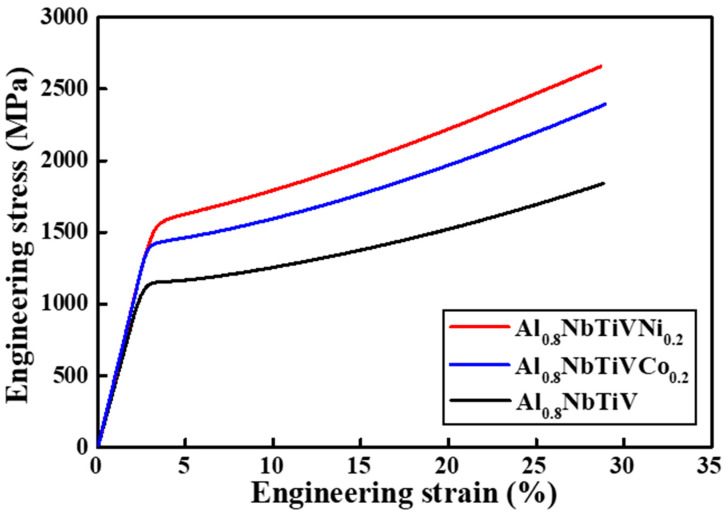
Stress–strain curves of as-cast Al_0.8_NbTiVM_0.2_ (M = Co, Ni) alloys at room temperature.

The compressive stress–strain curves of the as-cast Al_0.8_NbTiVCo_0.2_ and Al_0.8_NbTiVNi_0.2_ alloys at room temperature with a strain rate of 10^−3^ s^−1^ are shown in Figure 4.

For comparison, the stress–strain curve of the Al_0.8_NbTiV alloy is included in Figure 4, and the detailed mechanical property of present alloys are summarized in Table 2. The yield strength increases to about ~300 and ~350 MPa with addition of Co and Ni elements into the Al0.8NbTiV alloy. The enhanced yield strength can be anticipated due to the smaller atomic radius of Co and Ni in comparison with the atomic radii of other principal elements of the alloys, causing significant solid solution strengthening by severe lattice distortion [17].

### 3.3. Microstructure and Mechanical Properties of the Al0.8NbTiVM0.2 (M = Co, Ni) Alloys Heat-Treated at 1100 °C for 1 h

**Figure 5:**
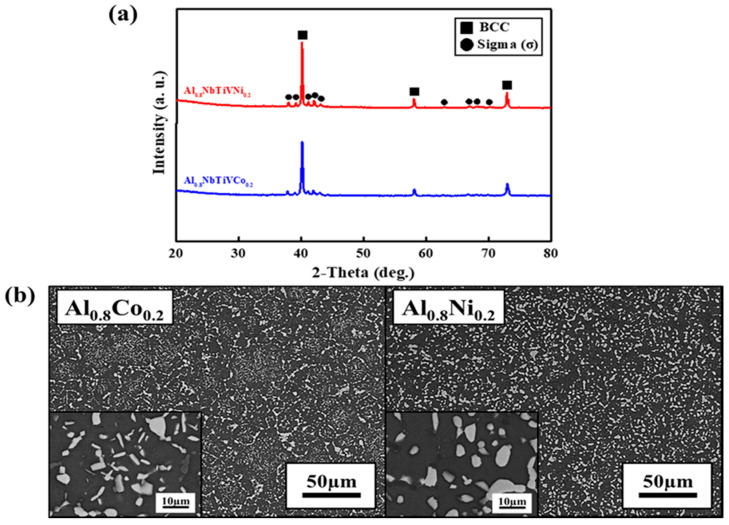
Microstructure of the Al_0.8_NbTiVM_0.2_ (M = Co, Ni) alloys heat-treated at 1100 °C for 1 h: (**a**) XRD patterns, (**b**) SEM-BSE images.

To confirm the single solid-solution phase stability and investigate microstructural evolution with mechanical property at equilibrium state, the Al_0.8_NbTiVM_0.2_ alloys were further subjected to heat treatment at 1100 °C for 1 h. Figure 5a shows the XRD patterns of the heat-treated Al_0.8_NbTiVCo_0.2_ and Al_0.8_NbTiVNi_0.2_ alloys at 1100 °C for 1 h. The diffraction peaks for the two alloys present are identified as a mixture of BCC phase and sigma phase (σ) with a tetragonal structure (the space group 136) [24,25]. The lattice constant of the BCC phase in the Al_0.8_NbTiVCo_0.2_ and Al_0.8_NbTiVNi_0.2_ alloys are estimated to be 0.31722 and 0.31760 nm respectively, which is similar to that of the as-cast alloys. The lattice constants of the sigma phase are a = 0.9638 and 0.9582 nm and c = 0.5682 and 0.5702 nm, respectively. Figure 5b shows the SEM image of the Al_0.8_NbTiVCo_0.2_ and Al_0.8_NbTiVNi_0.2_ alloys heat-treated at 1100 °C for 1 h.

Both alloys exhibit a similar microstructure, which consists of a homogeneously distributed secondary phase (particles) within the BCC matrix. Moreover, the distributed particles have two different contrasts in SEM-BSE images for both alloys, i.e., bright-contrast precipitates (major) and a small amount of dark-contrast precipitates. To confirm and identify the chemical compositions of precipitates, the point-EDS, line-EDS, and EDS-mapping analyses were performed (not shown). The bright contrast precipitates are enriched in Nb element, while the Ni and Co are more preferentially distributed in dark-contrast precipitates. According to the XRD results (Figure 5a), the two distributed precipitates are considered as the sigma (σ) phases. These results imply that Co and Ni are dissolved in dark precipitates instead of Nb. Hence, the bright precipitates can be Nb-rich sigma phase, whereas dark precipitates can be Co- and Ni-rich sigma phase. Therefore, Al_0.8_NbTiVCo_0.2_ and Al_0.8_NbTiVNi_0.2_ HEAs consist of BCC matrix with sigma precipitations, which contain different principal elements. The detailed chemical compositions of the composed phases for the two present alloys are summarized in Table 4.

The sigma phases have a crystal structure of tetragonal structure (the space group 136), which are often formed in binary and ternary alloy systems, such as Ni-V, Co-V, Nb-Al, Al-Nb-Ti, Al-Nb-V, Al-Nb-Ni, Al-Nb-Co, Nb-Ni-V, Co-Nb-V, and Ni-Ti-V [26,27,28,29,30,31,32,33,34]. According to the constitutive elements and the composition of the sigma phases for present alloys, the atomic structure of possible sigma phase is believed to be Al(Nb, Co)_2_ and Al(Nb, Ni)_2_ phases, since sigma phases in current alloys are enriched with Nb, Al, Ni, and Co. Note that the AlNb_2_ sigma phase also could contain ~11.4 at.% of Co, ~30 at.% of Ti, ~20 at.% of V, or ~10 at.% of Ni as solute elements [18,31,32]. Furthermore, the formation of AlNb_2_ sigma phase was often observed in Al-Nb-Ti-V HEAs at temperatures below 1200 °C, but the fraction was very limited [17]. In the present case, however, the addition of the Co and Ni results in the formation of a high fraction of the sigma phase, which could suggest that addition of Co and Ni efficiently stimulates the formation of Al(Nb, Co)_2_ and Al(Nb, Ni)_2_ sigma phases.

**Figure 6:**
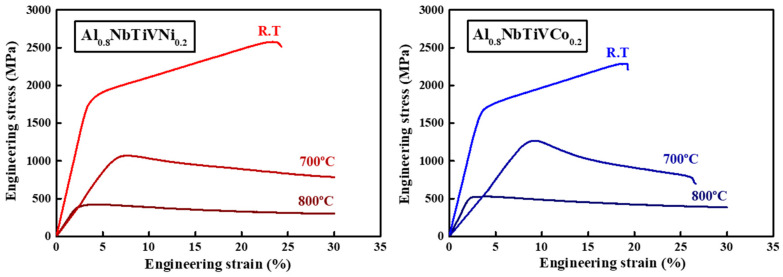
Stress–strain curves of the Al_0.8_NbTiVM_0.2_ (M = Co, Ni) alloys heat-treated at 1100 °C for 1 h at elevated temperatures.

The elevated temperature compressive tests were performed up to 800 °C at a strain rate of 1 × 10^−3^ s^−1^ for the heat-treated Al_0.8_NbTiVCo_0.2_ and Al_0.8_NbTiVNi_0.2_ alloys, as shown in Figure 6.

The homogeneously distributed sigma (σ) phase leads to a further improvement of the room-temperature yield strength, from 1473 to 1645 MPa for Al_0.8_NbTiVCo_0.2_ and 1510 to 1723 MPa for Al_0.8_NbTiVNi_0.2_, and a reduction of the room-temperature plastic strain. As the temperature increases, the yield strength of the heat-treated Al_0.8_NbTiVM_0.2_ (M = Co, Ni) alloys gradually decrease, from 1645 and 1723 MPa (RT) to 507 and 378 MPa (800 °C) for Al_0.8_NbTiVCo_0.2_ and Al_0.8_NbTiVNi_0.2_ alloys, respectively. The heat-treated Al_0.8_NbTiVCo_0.2_ alloy showed less plasticity than the Al_0.8_NbTiVNi_0.2_ alloy at 700 °C. In contrast to the deformation at room temperature, the stress–strain curves of the alloys at 700 and 800 °C indicated pronounced softening during plastic deformation. The sharp decrease of the strength at 800 °C is believed to be associated with the thermal softening of the sigma phase [18]. The detailed high-temperature mechanical properties of the current alloys are listed in Table 5.

The aim of the current work was to develop and design new RHEAs, which have low density and high strength at elevated temperature for high-temperature applications. The density of the Al_0.8_NbTiVM_0.2_ (M = Co, Ni) alloys was measured to be approximately 5.84 g cm^−3^, which is slightly larger density than the Al_0.8_NbTiV alloy (5.61 g cm^−3^) due to the addition of Co and Ni. However, this value is much lower than conventional high-temperature materials, such as Inconel 718 (8.22 g cm^−3^) [35] and RHEAs. Most reported RHEAs possess high density of 6–13 g cm^−3^, i.e., NbTiVZr and CrNbTiVZr alloys are 6.52 and 6.57 g cm^−3^ [23], respectively. The measured density of Al_0.8_NbTiVM_0.2_ (M = Co, Ni) alloys is summarized in Table 2. The values of measured densities are highlighted in bold.

**Figure 7:**
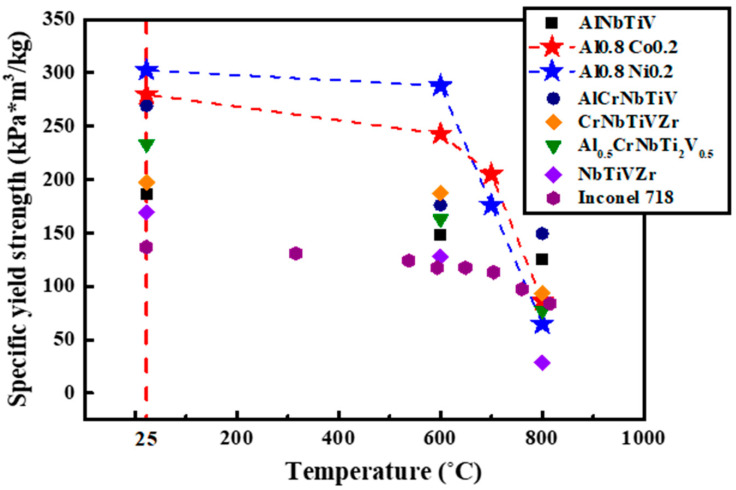
Specific yield strength of the Al_0.8_NbTiVM_0.2_ (M = Co, Ni) alloys, AlNbTiV [19], AlCrNbTiV [17], CrNbTiVZr [23], NbTiZrV [23], and Al_0.5_CrNbTi_2_V_0.5_ [36] HEAs and Inconel 718 [35].

With the consideration of low density in the present alloys, Figure 7 shows specific yield strength (SYS), yield strength/density, of the heat-treated Al_0.8_NbTiVM_0.2_ (M = Co, Ni) at elevated temperatures.

For the comparison, the SYS of the AlNbTiV, AlCrNbTiV, CrNbTiVZr, NbTiZrV, and Al_0.5_CrNbTi_2_V_0.5_ HEAs and Inconel 718 are also included [17,19,23,36]. Note that the SYS of the Inconel 718 alloy was obtained in tension. The addition of Co or Ni into the Al_0.8_NbTiV alloy results in the increase of the SYS as compared to the AlNbTiV HEA. It is observed that the SYS of the Al_0.8_NbTiVCo_0.2_ and Al_0.8_NbTiVNi_0.2_ alloys are higher than other alloys at temperatures up to 700 °C, while further increase of the temperatures induces a dramatic decrease of the SYS, to 86.65 and 64.79 kPa*m^3^/kg, respectively. Compared to Inconel 718, which is widely used for high-temperature applications, the present Al_0.8_NbTiVM_0.2_ (M = Co, Ni) alloys possess higher yield strength at elevated temperatures up to 700 °C.

In the present study, the addition of Co or Ni stimulates the homogeneously distributed sigma precipitates in the BCC solid-solution matrix, and it was demonstrated that the sigma precipitate plays a crucial role in the improved yield strength at room and elevated temperatures. There have been extensive efforts to improve the yield strength of RHEAs by controlling the size, volume fraction, and the feature of precipitates’ distribution [18,36,37], and it has been reported that the homogeneous distribution of fine precipitates is effective to improve the strength [37] compared to other control factors. As observed in the current case, the uniformly dispersed sigma precipitates in the Al_0.8_NbTiVM_0.2_ (M = Co, Ni) alloys are believed to improve the yield strength at room and high temperatures.

## 4. Conclusions

In this study, Co and Ni elements were added into Al_0.8_NbTiV alloy, which shows the best combination of strength and plasticity among a series of the single-phase AlxNbTiV alloys. To develop the precipitation-hardened light-weight RHEAs, the microstructure and mechanical properties have been systematically investigated for the presented newly designed RHEAs, as listed below:

- The AlxNbTiV (X = 0, 0.4, 0.8, 1) alloys in as-cast condition have single body-centered-cubic (BCC) structure. As the Al content increases up to 0.8, the yield strength is gradually enhanced to 1142 MPa without apparent reduction of the plasticity. Thus, the Al_0.8_NbTiV RHEA was selected for designing new precipitation-hardened light-weight RHEAs by addition of minor Co and Ni elements.
- The as-cast Al_0.8_NbTiVM_0.2_ (M = Co, Ni) alloys contain a single BCC phase. The yield strength of the as-cast Al_0.8_NbTiVM_0.2_ (M = Co, Ni) alloys at room temperature is higher than the Al_0.8_NbTiV alloy, which is due to the smaller atomic radius of Co and Ni in comparison with the atomic radii of other principal elements of the alloys, leading to significant solid-solution strengthening by severe lattice distortion.
- The heat treatment at 1100 °C for 1 h on the Al_0.8_NbTiVM_0.2_ (M = Co, Ni) alloys results in the formation of the sigma particles within the BCC matrix. The formation and homogeneous distribution of sigma particles plays a critical role in enhancement of yield strength in heat-treated Al_0.8_NbTiVM_0.2_ (M = Co, Ni) alloys. Moreover, the high-temperature compression tests (700 and 800 °C) were conducted for the Al_0.8_NbTiVM_0.2_ (M = Co, Ni), and the comparison of the temperature dependence of yield strength was made via the specific yield strength (yield strength/density). The specific yield strength of the Al_0.8_NbTiVM_0.2_ (M = Co, Ni) alloys is superior to single-phase AlNbTiV and NbTiVZr RHEA alloys and conventional Ni-based superalloy (Inconel 718 alloy) at elevated temperatures up to 700 °C.

## Figures and Tables

**Table 1:** Nominal compositions of the AlxNbTiV (X = 0, 0.4, 0.8, and 1.0) and Al_0.8_NbTiVM_0.2_ (M = Co, Ni) alloys.

Alloys	Atomic Percent (at.%)					
	Al	Ti	Nb	V	Co	Ni
NbTiV	-	33.33	33.33	33.33	-	-
Al_0.4_NbTiV	11.8	29.4	29.4	29.4	-	-
Al_0.8_NbTiV	21.1	26.3	26.3	26.3	-	-
AlNbTiV	25	25	25	25	-	-
Al_0.8_NbTiVCo_0.2_	20	25	25	25	5	-
Al_0.8_NbTiVNi_0.2_	20	25	25	25	-	5

**Table 2:** Compression mechanical properties, lattice constants (a) of BCC solid-solution phase, and density of the AlXNbTiV (X = 0, 0.4, 0.8, 1) and Al_0.8_NbTiVM_0.2_ (M = Co, Ni) alloys at room temperature.

Alloy	Condition	σ_0.2_ (MPa)	ε (%)	a (nm)	ρ_mix_ (g cm^−3^)	ρ_exp_ (g cm^−3^)
NbTiV	As-cast	790 ± 32	>30	0.32122 ± 0.00005	6.40	-
Al_0.4_NbTiV	As-cast	928 ± 12	>30	0.32036 ± 0.00009	5.96	-
Al_0.8_NbTiV	As-cast	1133 ± 72	>30	0.31940 ± 0.0001	5.61	-
AlNbTiV	As-cast	1379 ± 47	20.9 ± 4.9	0.31846 ± 0.00002	5.47	5.59
Al_0.8_NbTiV Co_0.2_	As-cast	1473 ± 109	>30	0.31742 ± 0.00005	5.73	5.841 ± 0.013
	Heat treatment	1645 ± 60	14.1 ± 0.8	0.31722 ± 0.0002	-	5.848 ± 0.042
Al_0.8_NbTiV Ni_0.2_	As-cast	1510 ± 13	>30	0.31738 ± 0.00006	5.73	5.841 ± 0.007
	Heat treatment	1723 ± 14	17.7 ± 2.4	0.31760 ± 0.00007	-	5.840 ± 0.051

**Table 3:** SEM-EDS composition and elemental distribution analyses of the as-cast Al_X_NbTiV (X = 0, 0.4, 0.8, 1) alloys.

Materials		Atomic Percent (at.%)			
		Elements			
Alloys	Designation	Ti	Nb	V	Al
NbTiV	Dendrite	32.3 ± 0.3	40.0 ± 0.3	27.7 ± 0.4	-
	Inter-dendrite	41.2 ± 0.5	18.1 ± 0.7	40.7 ± 0.3	-
Alloys	Designation	Ti	Nb	V	Al
Al0.4NbTiV	Dendrite	29.8 ± 0.4	32.3 ± 0.6	27.4 ± 0.2	10.5 ± 0.2
	Inter-dendrite	34.4 ± 0.6	21.9 ± 0.7	31.8 ± 0.5	11.9 ± 0.2
Alloys	Designation	Ti	Nb	V	Al
Al0.8NbTiV	Dendrite	27.7 ± 0.6	29.6 ± 0.3	25.2 ± 0.2	17.5 ± 0.5
	Inter-dendrite	30.6 ± 0.7	21.7 ± 8	27.4 ± 0.5	20.3 ± 0.8
Alloys	Designation	Ti	Nb	V	Al
AlNbTiV	Dendrite	24.4 ± 0.4	28.0 ± 0.2	26.2 ± 0.2	21.4 ± 0.6
	Inter-dendrite	29.5 ± 0.7	20.8 ± 0.8	25.5 ± 0.5	24.2 ± 0.5

**Table 4:** SEM-EDS composition and elemental distribution analyses of heat-treated Al_0.8_NbTiVM_0.2_ (M = Co, Ni) at 1100 °C for 1 h.

Materials		Atomic Percent (at.%)				
		Elements				
Alloys	Designation	Ti	Nb	V	Al	Co
Al_0.8_NbTiVCo_0.2_	Matrix	28.4 ± 1.0	23.0 ± 0.7	26.3 ± 0.4	17.4 ± 0.5	4.9 ± 0.5
	Nb-rich Sigma phase	20.5 ± 0.4	36.6 ± 0.4	15.7 ± 0.2	22.0 ± 0.3	5.2 ± 0.2
	Co-rich Sigma phase	20.1 ± 0.7	20.2 ± 0.3	20.2 ± 0.7	19.8 ± 0.4	19.7 ± 1.1
Alloys	Designation	Ti	Nb	V	Al	Ni
Al_0.8_NbTiVNi_0.2_	Matrix	29.0 ± 1.0	23.1 ± 1.0	27.0 ± 0.3	17.0 ± 0.5	3.9 ± 0.4
	Nb-rich Sigma phase	22.2 ± 0.4	34.5 ± 0.5	16.3 ± 0.4	21.2 ± 0.6	5.7 ± 0.4
	Ni-rich Sigma phase	23.1 ± 0.2	22.0 ± 0.8	12.0 ± 0.3	23.2 ± 0.3	19.6 ± 1.1

**Table 5:** Compression mechanical properties of the heat-treated Al0.8NbTiVM0.2 (M = Co, Ni) alloys at elevated temperatures.

Alloys	Al_0.8_NbTiVCo_0.2_		Al_0.8_NbTiVNi_0.2_	
Temperature	σ_0.2_ (MPa)	ε_p_ (%)	σ_0.2_ (MPa)	ε_p_ (%)
Room Temp.	1645	14.1	1723	17.7
700 °C	1198	21	1028	>25
800 °C	507	>28	378	>28

## Data Availability

Data is contained within the article.
